# Unpacking the mechanisms of self-regulated learning: How motivation translates into reading benefits for EFL adolescents

**DOI:** 10.3389/fpsyg.2026.1747968

**Published:** 2026-03-31

**Authors:** Xixi Yang, Yupei Zhang, Qi Nie

**Affiliations:** 1 English Department, Jiangxi Agricultural University, Nanchang, China; 2 Nanchang Middle School Education Group High-tech Campus, Nanchang, China

**Keywords:** reading motivation, self-regulated learning, reading engagement, metacognition, reading strategy, perceived benefits of reading, adolescent EFL learners, mediation

## Abstract

Understanding how motivation translates into learning outcomes is central to improving adolescent EFL reading development. While self-regulated learning is posited as a key mechanism, the distinct roles of its subcomponents—reading engagement, metacognition, and reading strategies—remain underexplored, particularly in specific instructional contexts. This study investigated the structural relationships in a multidimensional model of self-regulated reading among 203 Chinese middle school EFL learners. A questionnaire measuring reading motivation, reading engagement, metacognition, reading strategies, and perceived reading benefits was administered, and the data were analyzed using structural equation modeling (SEM). The analysis confirmed the validity of a second-order model in which self-regulated learning served as a higher-order construct encompassing reading engagement, metacognition, and reading strategies as first-order dimensions. The key finding revealed that the influence of reading motivation on perceived reading benefits was fully mediated by self-regulated learning. Subsequent analysis of the three self-regulatory dimensions indicated that all three loaded strongly onto the higher-order construct, with metacognition showing a slightly higher loading compared to engagement and strategy use. The uniformly high loadings across all three dimensions underscore that effective self-regulated reading requires the coordinated deployment of all three processes. These findings highlight the importance of fostering an integrated system of engagement, metacognition, and strategy use in adolescent EFL reading instruction.

## 1 Introduction

For adolescents worldwide, mastering reading in English as a Foreign Language (EFL) is a critical yet complex academic imperative. Previous research has shown that motivation and learning strategy use can vary depending on the subject and classroom context (Susiani et al., 2022). Studies have also demonstrated that motivation can influence reading achievement through engagement in general student populations (Guthrie et al., 2013; Klauda and Guthrie, 2015), that readers at different proficiency levels employ reading strategies differently (Denton et al., 2015), and that metacognitive strategies mediate the relationship between motivation and reading achievement (Jang et al., 2025), with its effects varying across learner populations (Kaya et al., 2022; Wei et al., 2025). While educators and researchers widely acknowledge the importance of motivational, strategic, and self-regulatory factors (Guthrie and Wigfield, 2000; Cheema, 2018), a pivotal question remains inadequately addressed: how do these elements interact dynamically to drive meaningful reading outcomes? Limited work has examined how these mechanisms operate collectively within the distinct context of adolescent EFL reading.

This research aims to illuminate how motivation translates into meaningful reading benefits by examining the mediating roles of engagement, metacognition, and strategy use. The findings offer teachers a practical framework for moving beyond motivation-building alone: by understanding which self-regulatory processes matter most, educators can design instruction that systematically cultivates not just motivated readers, but self-regulated learners who recognize and value their own growth.

## 2 Literature review

### 2.1 Reading motivation (RM)

Rooted in Self-Determination Theory (Deci and Ryan, 1985), reading motivation refers to the qualitative drive to read, encompassing an individual’s goals, values, and beliefs, and is typically differentiated into intrinsic and extrinsic facets (Guthrie and Wigfield, 2000). Research further reveals a more complex, multidimensional construct, with identified dimensions including curiosity, involvement, and social goals (Schiefele et al., 2012). The development of measurement tools reflects this complexity, though a noted challenge is the creation of reliable instruments that adequately capture these multiple dimensions across different age groups (Davis et al., 2018).

The influence of motivation on reading achievement is primarily mediated through behavioral engagement. Compelling evidence indicates that intrinsic reading motivation enhances higher-order reading comprehension by increasing the amount of reading, a factor that acts as a full mediator. Conversely, extrinsic motivation can exert direct and indirect negative effects on comprehension, often by reducing reading engagement (Schaffner et al., 2013; Areepattamannil et al., 2011). Furthermore, specific motivational dimensions differentially predict skills such as comprehension and summarization (McGeown et al., 2015), while positive reading habits, particularly fiction reading, serve as an independent behavioral predictor of competence (McGeown et al., 2015).

This motivation-achievement relationship is moderated by factors at multiple levels. Macro-level analyses show that a country’s overall academic performance can moderate the association, with the positive link between reading enjoyment and achievement reversing in lower-performing countries (Cheema, 2018). At the individual and group level, significant moderators include reading proficiency, gender, and socioeconomic status (SES). Struggling and proficient readers differ markedly across motivational dimensions (Wolters et al., 2014). Critically, a significant interaction exists between gender and SES, where lower family SES exacerbates motivational declines for boys, a gap that widens from middle elementary school onward (Becker and McElvany, 2018). Longitudinal studies confirm that early intrinsic motivation and engagement predict later reading achievement (Froiland and Oros, 2014).

In terms of educational practice, key motivational barriers for students—particularly boys—include lack of confidence, time constraints, perceived effort, and negative social perceptions (Senn, 2012; Wilkinson et al., 2020). In response, evidence-based strategies emphasize providing student choice in text selection and dedicating class time to independent reading and collaborative discussion (e.g., literature circles). These approaches effectively enhance reading self-concept, value, and positive experiences (Allred and Cena, 2020; Wilkinson et al., 2020).

### 2.2 Reading engagement (RE)

Reading engagement (RE) is recognized as a critical multifaceted construct in literacy development. Its conceptualization underscores the depth and quality of a learner’s involvement with text. Traditionally, engagement has been viewed through behavioral, affective, and cognitive lenses. Recent research expands this view to explicitly include a social dimension (e.g., sharing and discussing books), resulting in a comprehensive four-dimensional framework encompassing behavioral, cognitive, affective, and social aspects (McGeown and Smith, 2024). This evolution in theory reflects a growing understanding that meaningful engagement in reading is not merely an individual cognitive act but is enriched by social interaction and personal connection to the material (Ivey and Johnston, 2013).

Research on RE highlights its multifaceted nature and pivotal role. RE is recognized as a critical multifaceted construct in literacy development. Its conceptualization has evolved to encompass behavioral, affective, cognitive, and social dimensions, underscoring the depth of quality involvement with text (McGeown and Smith, 2024). Robust longitudinal evidence confirms that affective and behavioral facets, such as intrinsic motivation and active participation, are significant predictors of reading achievement (Froiland and Oros, 2014). However, this positive influence is moderated by contextual and individual factors, being notably weaker among struggling readers, which suggests the constraining role of concurrent cognitive challenges (Klauda and Guthrie, 2015).

Alarmingly, research consistently charts a decline in motivation and engagement from childhood through adolescence, a trend with discernible gender disparities (Brozo et al., 2014; Webber et al., 2023). In response, interventions ranging from school-wide cultural initiatives to targeted affective support have demonstrated efficacy in revitalizing engagement and its outcomes (Cockroft and Atkinson, 2017; Guthrie et al., 2013; Webber et al., 2023).

### 2.3 Metacognition (MC)

Grounded in Flavell’s (1979) seminal definition of metacognition as “thinking about one’s own thinking” and the self-regulation of cognitive processes, the term here refers to the strategic awareness and control learners exert over their learning activities.

Empirical evidence consistently underscores the pivotal role of MC in academic achievement. Large-scale studies confirm a positive correlation between MC strategy use and performance in reading, mathematics, and science (Chiu et al., 2007; Dent and Koenka, 2016), an effect that extends to digital reading (Lim and Jung, 2019) and is evident across diverse educational contexts (Areepattamannil, 2014).

However, this effect is not uniform and is moderated by learner characteristics. For instance, procedural MC predicts achievement in gifted students beyond intelligence (Tibken et al., 2022), while both linguistic and metacognitive knowledge explain comprehension levels in low-achievers (Trapman et al., 2017). Furthermore, students of different proficiency levels demonstrate varying frequencies of metacognitive strategy use (Wei et al., 2025). The relationship is typically stronger for non-immigrant students than for immigrants (Kaya et al., 2022), and the pathway from motivation to achievement varies by language background—being fully mediated by metacognitive strategies for some multilingual learners (Jang et al., 2025).

A key insight is that MC often acts as a critical mediator or conduit. It translates motivation into achievement (Jang et al., 2025) and is part of a broader motivational-strategic-performance pathway (Yau, 2022). It also mediates the relationship between other factors (e.g., social media use) and digital literacy (Chen et al., 2021). Its function is distinct from cognitive strategies, being more effective for developing problem-solving abilities, whereas cognitive strategies more directly aid reading comprehension (Ghahari and Basanjideh, 2015). These mechanisms are sensitive to context; in collective cultures, peer use of metacognitive strategies is more strongly linked to achievement than individual use (Chiu et al., 2007).

Instructional interventions successfully leverage this understanding. Professional development can enhance teachers’ metacognitive support, improving student outcomes (Greenleaf et al., 2011), and targeted digital scaffolding can boost comprehension, especially for below-average readers (Beek et al., 2019).

Underpinning this research is the recognition of metacognition as a multifaceted construct. The critical distinction between declarative knowledge and procedural skills (Tibken et al., 2022), together with the finding that metacognition’s correlation with achievement is moderated by measurement and contextual factors (Dent and Koenka, 2016), highlights the need for precise conceptualization in future work.

### 2.4 Reading strategy (RS)

A substantial body of research confirms that systematic instruction in reading strategies has a positive effect on enhancing adolescents’ reading comprehension and motivation (Cantrell et al., 2016; Shih and Reynolds, 2018). However, the efficacy of such interventions is notably complex and moderated by multiple factors.

Firstly, the cognitive and metacognitive foundations of individual learners are crucial prerequisites for the success of strategy interventions. Vocabulary knowledge constitutes a fundamental constraint. Low vocabulary can lead to cognitive overload, thereby attenuating the effectiveness of strategy instruction; this indicates that vocabulary is a prerequisite for the successful application of strategies (Okkinga et al., 2023). Concurrently, systematic differences exist in learners’ patterns of strategy use. Proficient readers employ high-level evaluative, integrative, and self-regulatory strategies more frequently than struggling readers (Cantrell and Carter, 2009; Denton et al., 2015), who may rely more on basic support strategies. These differences are associated not only with reading proficiency but also with grade level and gender: females typically report higher frequency of strategy use across all types, and strategy use tends to increase with grade level (Denton et al., 2015). Furthermore, learners’ attributions for success in reading—attributing success to ability rather than effort—are linked to higher reading skills, suggesting a deep-seated connection between motivational beliefs and strategy use (Frijters et al., 2018).

Secondly, the effectiveness of strategies varies dynamically with the complexity of the reading task and context. For single-text reading comprehension, explicit strategy instruction (e.g., incorporating goal setting, think-aloud protocols) has been shown to promote diversified strategy use and metacognitive awareness, although its effects are inconsistent across individuals, highlighting the need for personalized guidance (Salehomoum, 2022; Shih and Reynolds, 2018). When the task escalates to multiple-text reading or online information seeking, the required set of strategies becomes more complex. Research indicates that beyond foundational word recognition skills, higher-order strategies such as non-linear reading, synthesis, and evaluation of information, along with domain-specific reading self-efficacy, emerge as unique predictors of success in multi-text comprehension (Coiro, 2011). In such contexts, higher-order strategies can even partially compensate for a lack of topic-specific prior knowledge (Coiro, 2011).

Finally, a developmental perspective reveals that strategic competency is rooted in long-term reading trajectories. From early childhood through adolescence, a bidirectional relationship exists between reading skills (e.g., fluency) and reading practice (volume of independent reading): early skills foster reading engagement, and accumulated practice, in turn, further drives competency development in later stages (Bergen et al., 2021). More profound influences can be traced back to the preschool period. Vocabulary size, early literacy skills, and even oral narrative ability in early childhood significantly predict reading comprehension in adolescence, providing a rationale for fostering the foundational language and cognitive skills requisite for strategy use at an earlier stage (Suggate et al., 2018).

In summary, the research focus has shifted from demonstrating the efficacy of strategy instruction to a deeper investigation of the conditions and boundaries of its effectiveness. Future pedagogical practices must adopt a differentiated and dynamic lens. When implementing strategy interventions, it is essential to concurrently assess and support students’ vocabulary foundation, motivational beliefs, and metacognitive awareness. Additionally, the design should be tailored according to the specific demands of the reading task (e.g., single vs. multiple texts, offline vs. online) and the characteristics of the target student population (e.g., different grade levels, varying proficiency levels) (Guerin and Murphy, 2015; Huang, 2024).

### 2.5 Perceived benefits of reading (PBR)

The positive outcomes of reading extend far beyond performance on standardized comprehension tests. A critical yet less quantified aspect is learners’ subjective perception of the value and benefits they derive from reading, herein termed Perceived Benefits of Reading (PBR). This construct is fundamentally grounded in expectancy-value theory (Wigfield and Eccles, 2000), encompassing the utility value of reading for future goals, the intrinsic value of enjoyment, and the attainment value of being a good reader. Adolescents themselves articulate this value, viewing reading as an essential skill for long-term well-being and career opportunities (Fletcher and Nicholas, 2016), and even report greater enjoyment (an intrinsic reward) during successful word learning (Bains et al., 2024).

Empirical research, particularly from adolescent perspectives, reveals a rich tapestry of perceived gains. These include cognitive and academic benefits, such as improved comprehension facilitated by explicit teaching and the development of positive academic reading attitudes (Fletcher and Nicholas, 2016; Jang and Ryoo, 2019). Importantly, social and affective benefits are highly salient: adolescents value reading for fostering meaningful discussions, critical exploration, and social connection (Merga et al., 2018; Webber et al., 2025). Furthermore, when supported through practices like sustained silent reading with teacher mentoring, students report transformative gains in reading habits, attitudes, and self-efficacy (Lee, 2011).

The perception of these multifaceted benefits is not merely an outcome but a key mechanism that fuels further engagement. Factors that enhance PBR—such as access to interesting books, autonomy in choice, and enjoyable social reading practices—are precisely those that motivate adolescents’ volitional reading (Webber et al., 2025; Merga, 2015). While qualitative studies have richly described these benefit dimensions, there remains a need for an integrated, quantitative approach to systematically measure PBR and examine its antecedents.

### 2.6 Self-regulatory processes as mediating mechanisms in reading outcomes

A growing body of research has positioned reading engagement, metacognitive strategies, and reading strategies as key mediating mechanisms linking motivational and cognitive antecedents to reading outcomes.

Engagement has been found to mediate the relationship between reading emotions and comprehension (Hamedi et al., 2020), between language proficiency and comprehension (Taboada et al., 2013), and between instructional approaches and reading outcomes (Wigfield et al., 2008). These findings collectively position engagement as an explanatory mechanism through which affective, linguistic, and instructional factors influence reading comprehension.

Metacognitive strategies mediate the relationship between reading motivation and reading achievement, though the pattern may vary across learner populations (Jang et al., 2025). Miyamoto et al. (2019) further demonstrated that metacognitive knowledge of strategy use, alongside reading amount, mediates the motivation-comprehension relationship. Specific strategy types, particularly problem-solving strategies, have been shown to predict both literal and higher-order comprehension (Ghaith and El-Sanyoura, 2019).

Reading strategies uniquely mediate the relationship between motivation and comprehension (Liao et al., 2022; Sanir et al., 2023), and also mediate the effects of cognitive variables such as reading fluency and vocabulary knowledge on comprehension (Völlinger et al., 2018). Strategies have been found to completely mediate the relationship between academic self-efficacy and comprehension (Inciso-Rojas and García Ampudia, 2022), and this mediating role appears robust across learners with and without learning disabilities (Sanir et al., 2023).

While each dimension has been studied separately, these processes are theoretically interconnected as components of self-regulated learning. However, limited research has examined how engagement, metacognition, and reading strategies collectively operate within an integrated framework to mediate the relationship between motivation and reading outcomes among adolescent EFL learners—a gap addressed by the present study.

### 2.7 Theoretical framework

This study is grounded in two complementary theoretical perspectives that together provide a comprehensive lens for understanding how motivation translates into reading outcomes through self-regulatory processes.

The Engagement Model of Reading Development (Guthrie and Wigfield, 2000) posits that reading comprehension is fostered when readers are engaged—a state defined as the joint functioning of motivational processes and cognitive strategies during reading (Wigfield et al., 2008). According to this model, engaged readers are both internally motivated and strategic, and effective instruction enhances comprehension to the extent that it fosters these engagement processes.

To capture the full complexity of self-regulated reading, we integrate insights from Self-Regulated Learning (SRL) theory (Zimmerman, 1989; Pintrich et al., 1993). SRL describes the extent to which learners are metacognitively, motivationally, and behaviorally active participants in their own learning process (Zimmerman, 1989). This theoretical perspective emphasizes that genuine self-regulated learning involves an integrated process where learners proactively employ strategies to achieve academic goals based on perceptions of self-efficacy. Importantly, SRL theory distinguishes three core, empirically separable dimensions: metacognitive control (planning, monitoring, evaluating), behavioral engagement (effort, persistence), and cognitive strategy use (rehearsal, elaboration, and organization) (Pintrich et al., 1993).

Integrating these frameworks, we propose that in the context of adolescent EFL reading, the three dimensions identified in SRL theory—reading engagement (RE), metacognition (MC), and reading strategies (RS)—represent the key processes through which motivation translates into outcomes. Moreover, consistent with the Engagement Model’s emphasis on the integrated functioning of these processes, we conceptualize RE, MC, and RS as three first-order dimensions of a higher-order self-regulated learning (SRL) construct. This integrated model posits that reading motivation (RM) drives the higher-order SRL construct, which in turn generates perceived benefits of reading (PBR).

This framework extends the Engagement Model in two important ways: by explicitly distinguishing metacognition as a separate dimension alongside engagement and strategies, and by integrating these three dimensions into a higher-order SRL construct that captures their shared variance and collective functioning. It also extends SRL theory by applying it specifically to the EFL reading context and by linking it to subjectively experienced yet educationally significant outcomes—students’ perceived benefits of reading.

### 2.8 Research gaps and research questions

Based on the synthesis of existing literature, this study addresses two interconnected gaps in understanding self-regulated EFL reading. First, while theoretical frameworks posit a multidimensional structure of self-regulated learning encompassing engagement, metacognition, and strategy use, limited evidence validates such an integrated model—particularly one incorporating reading motivation—among specific learner populations such as Chinese middle school EFL learners. Second, although prior research has separately suggested mediating roles for these self-regulatory dimensions in the motivation-achievement relationship, their collective functioning within an integrated higher-order framework and their relative contributions as indicators of self-regulated reading remain underexplored.

In light of these gaps, the present study addresses the following research questions.

Research Question 1 (RQ1): Does the multidimensional measurement model demonstrate adequate psychometric properties for Chinese middle school students?

Research Question 2 (RQ2): To what extent is the influence of reading motivation on perceived reading benefits direct versus indirect?

Research Question 3 (RQ3): If an indirect effect exists, what are the relative contributions of reading engagement, metacognition, and reading strategies in this mediating pathway?

The three research questions were examined through the following sequential analyses using structural equation modeling (SEM) in AMOS 23.0: (1) Confirmatory factor analysis (CFA) was performed to evaluate the psychometric properties (convergent and discriminant validity) and overall fit of the hypothesized measurement model (RQ1). (2) Path analysis within the SEM framework was used to test the structural relationships, specifically assessing the significance of both the direct path (reading motivation → reading benefits) and the indirect effect via the higher-order self-regulated reading construct (SRL), using a bootstrap procedure with 2,000 resamples (RQ2). (3) To further examine the role of the three self-regulatory dimensions within this mediated pathway, the standardized factor loadings of reading engagement, metacognition, and reading strategies onto the higher-order SRL factor were examined, providing insight into their relative contributions as indicators of the self-regulated reading construct (RQ3). This analytic sequence provides a comprehensive test of the proposed theoretical model.

## 3 The method

### 3.1 Participants

This study was conducted in a public secondary school located in a medium-sized city in Central China. The student population is drawn primarily from working-class and middle-class families, representing a typical socio-economic profile for non-affluent urban areas in China’s interior. The school follows the standard national curriculum and is non-selective in its admissions. The participants were 259 seventh-graders (aged 12–13) from five classes. The sample included 130 males (50.2%) and 129 females (49.8%). All participants had a minimum of 5 years of English language learning experience, as Grade 3 typically marks the onset of English instruction in Chinese public primary schools.

Consistent with the national curriculum standards for compulsory education in China, all participants received 3–4 English classes per week (45 min each), with 1–2 sessions specifically allocated to reading instruction—aligning with the common practice in most Chinese junior high schools. These reading classes focused on textbook-based passages (e.g., narrative stories, expository articles) and supplementary graded readers (Level 5–7), aiming to develop foundational skills such as vocabulary recognition, literal comprehension, and basic inferencing. Teachers typically integrated explicit strategy guidance (e.g., teaching students to identify topic sentences or connect text to prior knowledge) into these sessions, which is a core requirement of China’s English teaching syllabus for lower secondary education.

In terms of out-of-class reading, students were assigned 2–3 reading tasks weekly (e.g., completing comprehension exercises, writing short summaries), with an estimated weekly reading volume of 800–1,200 words—consistent with the average workload for seventh-graders in China, where reading practice is primarily structured and task-driven rather than voluntary.

### 3.2 The measurement tool

This study adopted a 5-point Likert scale questionnaire to measure participants’ attitudes and reading behaviors (1 = Strongly Disagree, 2 = Disagree, 3 = Neutral, 4 = Agree, 5 = Strongly Agree), facilitating systematic collection of quantitative data. The questionnaire comprised 44 items (see Appendix Table A) measuring five main constructs: reading motivation (RM), reading engagement (RE), metacognition (MC), reading strategies (RS), and perceived benefits of reading (PBR). The operational definitions, number of items, sample items, and sources for each construct are detailed in Table 1.

**Table 1:** Constructs and operational definition of variables.

Construct	Operational definition in this study	Items	Sample item(s)	Source/development	Response scale
RM	RM is operationalized as the composite of students’ intrinsic interest, perceived value, and self-efficacy regarding reading, contrasted with extrinsic motivational drives.	7 items including V1, V2, V3, V4, V5, V6, V7.	“I believe in myself that I can master English reading.”	Adapted from the Motivated Strategies for Learning Questionnaire (MSLQ; Pintrich et al., 1993).	5-point Likert scale (1=Strongly Disagree, 2=Disagree, 3=Neutral, 4=Agree, 5=Strongly Agree)
PBR	Perceived gains in (a) cognitive and metacognitive competencies, (b) social and collaborative skills, and (c) affective-motivational reinforcement.	8 items including V31, V38, V39, V40, V41, V42, V43, V44.	“English reading learning has improved my self-directed learning ability.”	Conceptualized based on Wigfield and Eccles (2000).	
RE	RE is operationalized as the quality of a learner’s active involvement in reading, manifested through sustained behavioral participation, positive emotional connection, deep cognitive and strategic investment, and interactive social participation with texts and peers. It functions as the vital link between reading motivation and reading outcomes.	11 items including V8, V10, V11, V12, V13, V14, V15, V27, V28, V29, V30.	“In English reading classes, when collaborating with classmates on teacher-assigned reading tasks, I actively join in division and discussions.”	Adapted from MSLQ.	
MC	MC is operationalized as learners’ strategic regulation of their reading comprehension processes. This encompasses their knowledge about reading strategies, the procedural skill to monitor and adjust their understanding in real-time, and the actual application of evaluative and integrative strategies to comprehend and critique texts.	7 items including V9, V32, V33, V34, V35, V36, V37.	“In the process of learning English reading, I often set goals and plans for my own learning.”	Adapted from MSLQ.	
RS	RS, the adaptive repertoire of mental procedures and techniques that readers deliberately employ to construct meaning from text, is operationalized along lower-order support strategies (e.g., vocabulary aid, rereading) and higher-order evaluative strategies (e.g., synthesis, critique, source evaluation).	11 items including V16, V17, V18, V19, V20, V21, V22, V23, V24, V25, V26.	“In English reading class, I have questioned the viewpoints or content presented in the article.” “When reading English, I guess the meanings of unknown words based on the context.”	Adapted from MSLQ. Adapted from Survey of Reading Strategies (SORS; Mokhtari and Sheorey, 2002).	

A total of 259 paper questionnaires were distributed to seventh-grade students during regular English classes at the end of the academic year, with all returned (initial response rate = 100%). To ensure data quality, returned questionnaires were rigorously screened for invalid responses: 55 were excluded due to highly consistent response patterns (e.g., straight-lining, indicating careless responding), and 1 was excluded due to partial missing data. For the remaining dataset, minor missing values (<5% of total data) were handled via mean substitution for the respective scale or dimension to maintain sample size and statistical power. After this screening and imputation process, 203 valid questionnaires were retained, resulting in an effective response rate of 78.38% (203/259).

## 4 Results

### 4.1 Initial hypothesized model

To address the research questions (see 2.8.), an initial structural model was proposed with RM as the independent variable, PBR as the dependent variable, and three parallel mediators: RE, MC, and RS. The operational definitions and measurement items for each construct are detailed in Table 1.

After estimating the initial model, several psychometric issues were identified, including poor model fit, construct misspecification and low factor loadings. The initial model exhibited inadequate fit to the data: *χ*^2^/df = 2.748 was acceptable, but CFI (0.741) and TLI (0.726) were well below the 0.90 criterion, while RMSEA (0.093) and SRMR (0.089) exceeded the recommended cutoffs of 0.08 and 0.08, respectively, (Hu and Bentler, 1999). These indices indicated that the hypothesized measurement structure required substantial revision. Besides, variable RM initially included extrinsic motivation (V4, V5) as indicators. However, empirical analysis revealed a negative correlation between extrinsic motivation items and the reading outcome (Schaffner et al., 2013), which contradicted the positive correlation pattern of intrinsic motivation and introduced internal inconsistency within the construct. Furthermore, the factor loadings for items V4 (−0.213), V5 (−0.144), V8 (0.412), V25 (0.370), and V26 (0.196) were well below the recommended threshold of 0.60, detrimentally affecting the overall model fit.

### 4.2 Model modification

Following the removal of V4, V5, V8, V25, and V26, the model was re-estimated. The results indicated that several other items continued to show inadequate factor loadings: V3 (0.529), V6 (0.518), V9 (0.568), V30 (0.575), and V31 (0.551). These items were also removed to further refine the measurement model, while ensuring that each latent factor still contained at least three indicators—a widely accepted minimum for model identification.

After addressing low factor loadings, modification indices were examined to identify sources of localized misfit. Based on both statistical evidence and theoretical justification, three within-construct residual correlations were sequentially introduced. e22↔e23 (RS, MI = 51.263): Both items (V22, V23) assess inferential reading strategies (prediction and author intent inference), sharing conceptual grounding in meaning-based reasoning. e6↔e7 (RM, MI = 42.002): Both items (V1, V2) capture the affective dimension of intrinsic motivation (joy and immersive experience). e43↔e44 (PBR, MI = 38.289): Both items (V43, V44) reflect affective gains from reading (confidence and interest), which are theoretically interrelated. With the inclusion of the three theoretically justified residual correlations above, the measurement model demonstrated improved fit: *χ*^2^/df = 2.681, CFI = 0.829, SRMR = 0.082, RMSEA = 0.091. Compared to the initial model (*χ*^2^/df = 2.748, CFI = 0.741, SRMR = 0.089, RMSEA = 0.093), these results indicate effective model refinement.

Then the revised first-order measurement model was assessed using confirmatory factor analysis (CFA). However, the model failed to meet the recommended criteria for construct validity. Several constructs exhibited suboptimal average variance extracted (AVE), with RM falling below the 0.50 threshold (AVE = 0.41). More critically, discriminant validity was not established for most construct pairs. According to established guidelines, discriminant validity is present when the shared variance within a construct (measured by AVE) exceeds its shared variance with any other construct (Hair et al., 2018). The square root of AVE for RM (0.758) was lower than its correlations with RE (0.921), RS (0.874), and MC (0.861). Similarly, the square root of AVE for RE (0.797), RS (0.712), and MC (0.640) were generally exceeded by their inter-correlations: RE–RS (0.805 > 0.712), RE–MC (0.793 > 0.640), and RS–MC (0.753 > 0.640). PBR also lacked discriminant validity with MC (0.798 > 0.731). These pervasive violations—particularly the high inter-correlations among RE, RS, and MC—indicate that these three dimensions are not empirically distinct, suggesting the presence of a higher-order construct accounting for their shared variance. This pattern aligns with theoretical frameworks conceptualizing engagement, metacognition, and strategy use as integrated facets of SRL (Zimmerman, 1989; Pintrich et al., 1993). Accordingly, a second-order factor model was specified, in which SRL serves as a higher-order latent variable with RE, MC, and RS as its first-order indicators.

Prior to establishing the final second-order model, two additional refinements were implemented based on modification indices and theoretical considerations.

A high modification index was observed for the cross-construct residual correlation between e36 (MC) and e46 (PBR), MI = 44.057. Closer examination revealed that item V37 (“I conduct self-assessments to evaluate my learning effectiveness and adjust my methods accordingly”) originally specified under MC shared substantial conceptual overlap with PBR items measuring perceived metacognitive gains, particularly V39 (“English reading learning has improved my self-directed learning ability”). After allowing these residuals to correlate, model fit improved marginally, and the MI value between e36 and e46 still remained high (30.42), indicating that the cross-loading issue persisted. Based on this theoretical overlap and the persistent high MI, V37 was re-specified as an indicator of PBR. Therefore, the problematic cross-construct residual correlation was resolved, and model fit improved notably.

Finally, a within-construct residual correlation between e23 and e24 (both within RS) was introduced (MI = 33.17). Both items assess inferential reading strategies: V22 measures prediction of story development, and V21 measures guessing word meanings from context. This correlation was theoretically justified given their shared focus on context-based inferential processing.

### 4.3 Final second-order model

The sample size (*N* = 203) is considered adequate for the proposed SEM analysis. Following Hair et al. (2018), sample sizes of 200 and larger are recommended as the number of variables and expected number of factors increases, which aligns with the complexity of the present measurement model. The final model includes 34 observed items measuring five first-order factors and one second-order factor. The cases-to-item ratio of approximately 5.97:1 meets the minimum requirement of 5:1 for exploratory purposes, and the strong factor loadings (all above 0.60) further support the stability of the measurement model.

**Figure 1:**
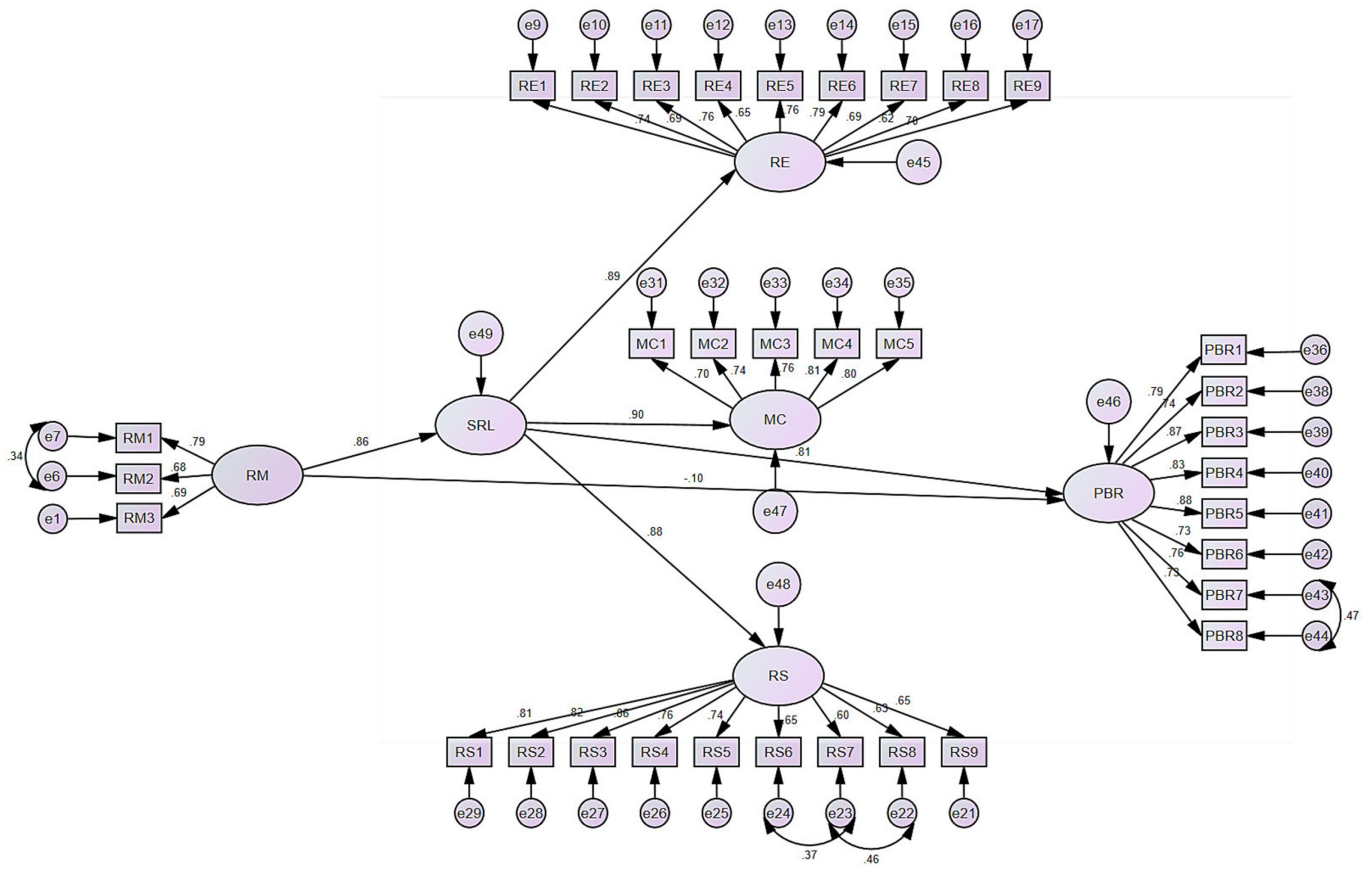
Structural equation model of the relationships among RM, SRL, and PBR.

The final second-order model (see Figure 1) demonstrated acceptable fit to the data: *χ*^2^/df = 2.616, CFI = 0.836, SRMR = 0.082, RMSEA = 0.089 (90% CI [0.084, 0.095]) (see Table 2). While the CFI fell slightly below the conventional threshold of 0.90, it is within the acceptable range for complex models with moderate sample size. The SRMR met the recommended criterion, and the RMSEA confidence interval did not exceed 0.10, indicating adequate model fit. The complete correspondence between each latent variable and its observed items in the final measurement model is provided in Appendix Table B.

**Table 2:** Goodness-of-fit indices for the final second-order model (*N* = 203).

Fit Index	Value	Criterion	Evaluation
χ^2^/df	2.616	<3 (good)	Good
CFI	0.836	>0.90 (good), >0.80 (acceptable)	Acceptable
TLI	0.822	>0.90 (good), >0.80 (acceptable)	Acceptable
RMSEA	0.089	<0.08 (good), <0.10 (acceptable)	Acceptable
RMSEA 90% CI	[0.084, 0.095]	Upper bound < 0.10	Met
SRMR	0.082	<0.08 (good), <0.10 (acceptable)	Good/Acceptable

Convergent validity was established for all constructs (see Table 3). The second-order factor SRL exhibited strong psychometric properties, with factor loadings of 0.905 (MC), 0.880 (RS), and 0.894 (RE), and an AVE of 0.798 and CR of 0.922, both exceeding the recommended thresholds of 0.50 for AVE and 0.70 for CR. All first-order factors also achieved satisfactory AVE and CR values: RM (AVE = 0.519, CR = 0.763), RE (AVE = 0.508, CR = 0.902), RS (AVE = 0.534, CR = 0.910), MC (AVE = 0.582, CR = 0.874), and PBR (AVE = 0.629, CR = 0.931). All factor loadings were significant at *p* < 0.001.

**Table 3:** Convergent validity.

Construct	Indicator	Standardized loading	AVE	CR
Second-order factor				
SRL	MC	0.905	0.798	0.922
	RS	0.880		
	RE	0.894		
			
First-order factor				
RM	V1	0.789	0.519	0.763
	V2	0.681		
	V7	0.686		
RE	V10	0.738	0.508	0.902
	V11	0.686		
	V12	0.759		
	V13	0.647		
	V14	0.762		
	V15	0.791		
	V27	0.695		
	V28	0.617		
	V29	0.699		
RS	V16	0.805	0.534	0.91
	V17	0.822		
	V18	0.864		
	V19	0.759		
	V20	0.740		
	V21	0.649		
	V22	0.601		
	V23	0.634		
	V24	0.654		
MC	V32	0.701	0.582	0.874
	V33	0.739		
	V34	0.764		
	V35	0.805		
	V36	0.800		
PBR	V37	0.786	0.629	0.931
	V38	0.742		
	V39	0.872		
	V40	0.833		
	V41	0.877		
	V42	0.732		
	V43	0.759		
	V44	0.726		

All factor loadings are standardized and significant at *p* < 0.001. AVE, average variance extracted; CR, composite reliability.

Discriminant validity was assessed using the Fornell-Larcker criterion (see Table 4). The square root of AVE for RM (0.720) was lower than its correlation with SRL (0.856), which is theoretically expected as RM serves as the antecedent of SRL in the structural model. PBR demonstrated satisfactory discriminant validity, with its square root of AVE (0.793) exceeding its correlations with RM (0.598) and SRL (0.729). According to Hair et al. (2018), in a reflective second-order model, the first-order factors “are now indicators of the second-order factor” and thus “are expected to move together (covary).” Consequently, when multiple first-order factors serve as indicators of a higher-order construct, the researcher “gives up the ability to test for relationships between these first-order factors and other key constructs.” Thus in the present study, the three first-order factors RE, RS, MC function as indicators of the second-order factor SRL; their inter-correlations are fully accounted for by the higher-order structure and do not require separate discriminant validity testing.

**Table 4:** Discriminant validity (Fornell–Larcker criterion).

Construct	RM	SRL	PBR
RM	**0.72**		
SRL	0.856	**0.893**	
PBR	0.598	0.729	**0.793**

Diagonal elements (bold) represent the square root of average AVE. Off-diagonal elements represent latent construct correlations.

Overall, the measurement model exhibited adequate psychometric properties, providing a solid foundation for subsequent structural path analysis.

### 4.4 Mediation analysis results

A bias-corrected bootstrap procedure with 2,000 resamples was conducted to test the mediating role of SRL in the relationship between RM and PBR. The results are summarized in Tables 5, 6.

**Table 5:** Bootstrap mediation results (standardized effects).

Effect	Path	Estimate	95% BC CI	*p*
Indirect effect	RM → SRL → PBR	0.698	[0.297, 1.645]	0.008
Direct effect	RM → PBR	−0.100	[−0.951, 0.432]	0.644
Total effect	RM → PBR	0.598	[0.416, 0.741]	0.001

BC CI, bias-corrected confidence interval (2,000 bootstrap samples).

**Table 6:** Standardized path coefficients for the structural model.

Path	Estimate	95% BC CI	*p*
RM → SRL	0.856	[0.738, 0.951]	0.001
SRL → PBR	0.815	[0.289, 1.608]	0.012
RM → PBR	−0.100	[−0.951, 0.432]	0.644
SRL → MC	0.905	[0.821, 0.963]	0.001
SRL → RS	0.880	[0.778, 0.945]	0.002
SRL → RE	0.894	[0.754, 0.955]	0.003

BC CI, bias-corrected confidence interval (2,000 bootstrap samples).

As shown in Table 5, the standardized indirect effect of RM on PBR via SRL was 0.698, with a 95% bias-corrected confidence interval of [0.297, 1.645]. Since the interval did not include zero, the indirect effect was statistically significant (*p* = 0.008). The direct effect of RM on PBR was not significant (*β* = −0.100, 95% CI [−0.951, 0.432], *p* = 0.644), while the total effect was significant (*β* = 0.598, 95% CI [0.416, 0.741], *p* = 0.001). These findings indicate that SRL fully mediates the relationship between RM and PBR, with the indirect effect accounting for the entirety of the significant total effect.

Table 6 presents the standardized path coefficients for the structural model. RM had a strong positive effect on SRL (*β* = 0.856, 95% CI [0.738, 0.951], *p* = 0.001), and SRL in turn had a significant positive effect on PBR (*β* = 0.815, 95% CI [0.289, 1.608], *p* = 0.012). The second-order factor SRL was well reflected by its three first-order dimensions, with all loadings exceeding 0.88 and statistically significant: MC (*β* = 0.905, 95% CI [0.821, 0.963], *p* = 0.001), RE (*β* = 0.894, 95% CI [0.754, 0.955], *p* = 0.003), and RS (*β* = 0.880, 95% CI [0.778, 0.945], *p* = 0.002). The uniformly high loadings confirm that engagement, metacognition, and strategy use are integral facets of self-regulated learning.

In summary, the results provide strong evidence that reading motivation enhances learners’ perceived reading benefits entirely through the enhancement of their self-regulated learning capacity.

## 5 Discussion

The present study examined the structural relationships among RM, SRL (conceptualized as a higher-order construct comprising RE, MC, and RS), and perceived PBR in a sample of Chinese middle school EFL learners. The findings offer nuanced insights into the mechanisms through which motivation translates into learning gains, addressing the specific research questions and enriching the existing literature.

### 5.1 Interpretation of key findings in relation to research questions

#### 5.1.1 Addressing RQ1: psychometric adequacy of the measurement model

The CFA results confirmed that the hypothesized second-order model of self-regulated learning is psychometrically sound for Chinese middle school EFL students. The model achieved acceptable fit across multiple indices, with *χ*^2^/df (2.616), CFI (0.836), SRMR (0.082), and RMSEA (0.089) (90% CI [0.084, 0.095]) all within or approaching recommended ranges for complex models with moderate sample size (Hu and Bentler, 1999). The satisfactory convergent validity across all constructs (AVEs ranging from 0.519 to 0.798, CRs from 0.763 to 0.931) further strengthens confidence in the measurement quality. Notably, the high AVE for SRL (0.798) indicates that the three first-order factors collectively capture the essence of self-regulated learning with minimal measurement error.

#### 5.1.2 Addressing RQ2: the mediated pathway from motivation to reading benefits

The finding that self-regulated learning fully mediates the relationship between reading motivation and perceived benefits carries several important theoretical implications.

First, this full mediation pattern provides strong empirical support for the behavioral mediation hypothesis articulated in prior motivation research. As Schaffner et al. (2013) demonstrated, intrinsic reading motivation enhances reading comprehension by increasing the amount of reading, which acts as a full mediator. The present findings extend this classic result by revealing that motivation operates through a broader suite of self-regulatory processes—engagement, metacognition, and strategy use—rather than merely through reading volume. Moreover, this mediated pathway holds for learners’ perceived benefits of reading, including gains in critical thinking, self-directed learning, and confidence. This extension is theoretically important because the perception of these multifaceted benefits is not merely an outcome but a key mechanism that fuels further engagement (Webber et al., 2025; Merga, 2015). By demonstrating that motivation operates through SRL to enhance PBR, the present study empirically validates this reciprocal relationship: self-regulated reading produces perceived gains that, in turn, are likely to sustain future motivational engagement.

Importantly, the complete mediation observed here also addresses a measurement challenge raised by Davis et al. (2018) regarding the difficulty of adequately capturing motivational dimensions across different age groups. The fact that motivation’s effects were fully transmitted through well-specified self-regulatory processes—rather than through a direct path—provides nomological validation for the RM construct. Motivation related strongly to self-regulatory processes (*β* = 0.856) and, through them, indirectly to perceived reading outcomes. This pattern of theoretically expected relationships suggests that the RM measure, despite the inherent challenges of assessing motivation in adolescent populations, was meaningfully operationalized.

Beyond this, the findings validate and extend the theoretical grounding of PBR in expectancy-value theory (Wigfield and Eccles, 2000). The significant indirect effect of RM on PBR via SRL suggests that motivation’s value components—intrinsic, utility, and attainment value—are realized through the self-regulatory processes that enable learners to translate their values into tangible gains. This aligns with the observation that adolescents view reading as an essential skill for long-term well-being and career opportunities (Fletcher and Nicholas, 2016)—a perception that likely emerges from accumulated experiences of successful, self-regulated reading.

Furthermore, the findings provide quantitative evidence that self-regulatory processes generate the specific benefits that adolescents themselves report valuing. Researches have highlighted that adolescents value reading for fostering meaningful discussions, critical exploration, and social connection (Merga et al., 2018; Webber et al., 2025). The present study demonstrates that these valued outcomes are systematically produced through self-regulated reading, with the social engagement captured in RE items measuring discussion and collaboration playing an integral role in this process.

#### 5.1.3 Addressing RQ3: roles of SRL sub-dimensions

RQ3 sought to examine the relative contributions of the three self-regulatory dimensions—RE, MC, and RS—within this mediating pathway.

The findings revealed that all three dimensions loaded strongly and comparably onto the higher-order SRL factor, with standardized loadings of 0.905 (MC), 0.894 (RE), and 0.880 (RS). The narrow range of these values (0.880–0.905) indicates that engagement, metacognition, and strategy use are not competing components but mutually reinforcing facets of an integrated self-regulatory system. This pattern provides empirical support for conceptualizing SRL as a coherent, multidimensional construct (Zimmerman, 1989; Pintrich et al., 1993), demonstrating that these dimensions cohere as manifestations of a shared underlying process rather than operating independently. The strong loading of RE (0.894) onto the higher-order SRL factor confirms that engagement is an integral component of the self-regulatory system, consistent with research demonstrating that affective and behavioral facets of engagement significantly predict reading achievement (Froiland and Oros, 2014). This finding aligns with contemporary frameworks that position engagement as a central rather than peripheral element of effective reading (McGeown and Smith, 2024). The particularly strong loading of MC (0.905) resonates with the key insight that metacognitive strategies often act as a critical mediator, translating motivation into achievement (Jang et al., 2025; Yau, 2022). Similarly, the strong loading of RS (0.880) onto the higher-order SRL factor indicates that strategy use is an integral component of the self-regulatory system. This finding is consistent with the well-documented relationship between strategy use and reading outcomes (Chiu et al., 2007; Dent and Koenka, 2016). It suggests that the strategies students employ are not isolated techniques but rather function within a broader self-regulatory framework that coordinates their deployment alongside engagement and metacognitive control.

The slight numerical differences among the loadings should not be overinterpreted. All three values fall within a narrow and excellent range, indicating that each dimension contributes substantially to the higher-order construct. The uniformly strong contributions across all three dimensions underscore that self-regulated reading is a multifaceted endeavor requiring the coordinated deployment of engagement, metacognition, and strategic processing.

### 5.2 Pedagogical implications

The findings of this study offer several actionable insights for EFL reading instruction, grounded in the specific behaviors and competencies captured by the measurement model.

To start with, the full mediation model underscores that cultivating motivation alone is insufficient; it must be accompanied by systematic development of self-regulatory competencies. While students may report joy and satisfaction in reading, find reading as engaging as watching an exciting movie, or feel fully immersed in texts, these motivational states do not automatically translate into learning gains. The present findings demonstrate that motivation’s benefits are entirely channeled through self-regulatory processes. Therefore, teachers should not assume that motivated students will naturally read effectively; instead, they must intentionally teach students how to plan, monitor, and evaluate their reading, how to deploy appropriate strategies, and how to sustain engagement even when texts are challenging.

Secondly, the integrated nature of the three self-regulatory dimensions calls for holistic instructional approaches that simultaneously address engagement, metacognition, and strategy use. The comparably strong loadings of RE, MC, and RS onto SRL indicate that these processes work in concert rather than in isolation. Classroom practices should therefore weave these components together in mutually reinforcing ways.

For example, teachers can design collaborative reading tasks that engage students in meaningful discussions while simultaneously activating metacognitive and strategic processes. When students share their thoughts, compare their own perspectives with those of peers, and actively join in discussions during collaborative tasks, they are not only behaviorally engaged but also exercising metacognitive awareness of their own and others’ understanding. To deepen this integration, teachers can structure discussions around open-ended questions that invite multiple possible answers and the integration of diverse perspectives, thereby promoting strategic thinking alongside social engagement.

This integrated approach can be further extended by embedding metacognitive and strategic elements into the same collaborative activities. Before discussions, teachers can guide students to set goals and plans for their learning and consider how they will approach the text—activating metacognitive planning while also preparing for engagement. During reading and discussion, students can practice controlling their attention and proactively seeking help when problems arise. At the same time, they can employ various reading strategies, including connecting prior knowledge with new information, drawing on knowledge from other subjects, organizing information using tables or mind maps, predicting story development, and inferring author intentions. After discussions, structured opportunities for reflection—such as identifying strengths and weaknesses in their learning experiences and comparing their approaches with those of others to refine their own methods—allow students to evaluate not only what they learned but also how effectively they engaged, which strategies served them well, and what they might adjust next time.

Finally, the finding that self-regulated reading generates the specific benefits that students themselves value provides a compelling rationale for making these benefits visible in the classroom. Students in this study reported gains in multiple areas: reading knowledge and skills, self-directed learning ability, information literacy, critical thinking, cooperation with others, confidence, interest, and the ability to conduct self-assessments to evaluate their learning effectiveness and adjust methods accordingly. These perceived benefits are not merely byproducts but are systematically produced through self-regulated reading. Teachers can leverage this by explicitly connecting classroom activities to these valued outcomes. For example, after collaborative discussions, teachers can highlight how sharing ideas and comparing perspectives contributed to deeper understanding and critical thinking. After strategy instruction, teachers can help students recognize how connecting prior knowledge, predicting story development, or inferring author intentions enhanced their comprehension and confidence. When students see that their efforts produce tangible benefits—greater confidence, sharper thinking, better collaboration, improved self-directed learning—they are more likely to sustain engagement, creating a positive feedback loop that reinforces self-regulated reading.

### 5.3 Limitations

This study has several limitations that should be considered when interpreting the findings.

One limitation concerns the reliance on self-reported data. While appropriate for capturing perceptual constructs such as motivation, engagement, and perceived benefits, this method may introduce common method bias and does not capture objective reading performance. Future research should employ multi-method assessments (e.g., combining self-reports with behavioral tasks, teacher ratings, or standardized reading comprehension tests) to complement and validate the self-reported measures.

A related methodological limitation involves the cross-sectional design and relatively modest sample size (*N* = 203). Despite the use of bootstrapping techniques, these factors limit causal inferences and parameter stability. Longitudinal or experimental designs with larger and more diverse samples are needed to establish the temporal precedence of the proposed mediation pathways and enhance the robustness of the findings.

Regarding generalizability, the sample was drawn from a specific population—Chinese middle school EFL learners from a public school in a medium-sized city in Central China. This may affect the transferability of the results to other age groups, proficiency levels, or cultural-linguistic contexts. Replication studies with more diverse populations are necessary to determine the extent to which the findings hold across different educational settings.

## 6 Conclusion

This study provides empirical evidence that for adolescent EFL learners, the pathway from reading motivation to meaningful learning outcomes is critically governed by learners’ capacity for self-regulation. By validating a second-order model of self-regulated reading, we confirm that motivation’s influence is fully mediated through integrated self-regulatory processes encompassing engagement, metacognition, and strategy use.

The most significant contribution of this research lies in its fine-grained dissection of this mediation mechanism. The findings reveal that all three self-regulatory dimensions—engagement, metacognition, and strategy use—function as an integrated system, with uniformly high loadings underscoring that effective self-regulated reading requires the coordinated deployment of all three processes rather than the dominance of any single component.

In summary, effective reading instruction is not merely about sparking interest—it is about building readers who can sustain that interest through purposeful action. The motivated student who lacks self-regulatory skills remains a spectator to learning; the truly empowered reader is one who can channel motivation into sustained engagement, metacognitive awareness, and strategic versatility. By designing instruction that weaves these three strands together, and by helping students recognize the tangible benefits such integrated effort produces, educators can cultivate not just better test-takers, but lifelong readers who read with curiosity, involvement, awareness, and strategic thinking.

## Data Availability

The original contributions presented in the study are included in the article/Supplementary material, further inquiries can be directed to the corresponding author.
