# Supplementary material for: Unpacking the mechanisms of self-regulated learning: How motivation translates into reading benefits for EFL adolescents
Source: Front Psychol. 2026 Mar 31;17:1747968. doi: 10.3389/fpsyg.2026.1747968 (PMC13076252; doi:10.3389/fpsyg.2026.1747968)
Supplement: Supplementary file 1 [file Supplementary_file_1.docx]

**Appendix**

**Table A.** Observed Item Details of the Questionnaire.

| Items | Content |
| --- | --- |
| V1 | I experience joy and satisfaction when reading English articles. |
| V2 | Reading English articles is like watching an exciting movie or novel. |
| V3 | English reading is valuable and meaningful to me. |
| V4 | I learn English reading because it holds significant weight in the middle school exam, rather than out of interest. |
| V5 | I study English reading because good grades can lead to rewards. |
| V6 | I believe in myself that I can master English reading. |
| V7 | When reading English articles, I can fully immerse myself and lose track of my surroundings. |
| V8 | Before reading English texts, I prepare thoroughly by looking up unfamiliar words. |
| V9 | When reading English articles, I usually have clear goals and questions in mind. |
| V10 | I actively participate in discussions and express my opinions. |
| V11 | In English reading class, I will actively answer questions posed by the teacher. |
| V12 | After class, I will discuss the questions in the article with the teacher or classmates. |
| V13 | I am willing to spend my spare time reading interesting English articles. |
| V14 | In English reading class, I use the learned vocabulary and sentence patterns to communicate with my peers. |
| V15 | In English reading class, I can use various English expressions to articulate my opinions. |
| V16 | In English reading class, I enjoy proposing multiple possible answers to the open-ended questions posed by the teacher. |
| V17 | In English reading class, I strive to integrate diverse perspectives before presenting my own opinions. |
| V18 | When reading in English, I connect prior knowledge with new information to enhance my understanding. |
| V19 | I draw on knowledge from other subjects to solve challenges encountered in English reading. |
| V20 | In English reading classes, I can organize what I’ve learned in tables or mind maps. |
| V21 | When reading English, I guess the meanings of unknown words based on the context. |
| V22 | When reading in English, I predict how the story will develop based on the article’s meaning. |
| V23 | When reading English, I infer the author’s intentions based on the meaning of the article. |
| V24 | When reading in English, I analyze the grammatical structures of complex sentences to aid my comprehension. |
| V25 | In English reading class, I have questioned the viewpoints or content presented in the article. |
| V26 | In English reading class, I have questioned inaccuracies in the teacher’s explanations. |
| V27 | In English reading class, I compare my thoughts with others’ views to enhance my understanding. |
| V28 | In English reading class, when the teacher asks me and my classmates to work together on a reading task, I actively participate in the division of labor and discussion. |
| V29 | In English reading class discussions, I share my thoughts with others. |
| V30 | In English reading class discussions, I listen carefully to others’ views and opinions. |
| V31 | In English reading class, collaborating with classmates helps us solve difficult problems. |
| V32 | In English reading class, I can control my attention to ensure high classroom efficiency. |
| V33 | When I can’t solve problems in English reading, I proactively ask teachers for help. |
| V34 | In the process of learning English reading, I often set goals and plans for my own learning. |
| V35 | Throughout my English reading journey, I compare my learning methods, ideas, and skills with those of others to refine my own approach. |
| V36 | Throughout my English reading journey, I often reflect on my learning experiences to identify my strengths and weaknesses. |
| V37 | During my English reading journey, I conduct self-assessments to evaluate my learning effectiveness and adjust my methods accordingly. |
| V38 | English reading learning has boosted my reading knowledge and skills. |
| V39 | English reading learning has improved my self-directed learning ability. |
| V40 | Studying English reading has sharpened my critical thinking skills. |
| V41 | Engaging with English reading has boosted my self-directed learning abilities. |
| V42 | English reading learning has improved my ability to cooperate with others. |
| V43 | English reading learning has increased my confidence in English learning. |
| V44 | Studying English reading has heightened my interest in the subject. |

**Table B.** Summary of Constructs and Corresponding Items in the Final Second-Order Model

| Construct | Indicator | Item Code |
| --- | --- | --- |
| Second-Order Factor | |  |
| Self-Regulated Learning (SRL) | RE (Reading Engagement) | — |
|  | MC (Metacognition) | — |
|  | RS (Reading Strategies) | — |
|  |  |  |
| First-Order Factors | |  |
| Reading Motivation (RM) | RM1 | V1 |
|  | RM2 | V2 |
|  | RM3 | V7 |
|  |  |  |
| Reading Engagement (RE) | RE1 | V10 |
|  | RE2 | V11 |
|  | RE3 | V12 |
|  | RE4 | V13 |
|  | RE5 | V14 |
|  | RE6 | V15 |
|  | RE7 | V27 |
|  | RE8 | V28 |
|  | RE9 | V29 |
|  |  |  |
| Metacognition (MC) | MC1 | V32 |
|  | MC2 | V33 |
|  | MC3 | V34 |
|  | MC4 | V35 |
|  | MC5 | V36 |
|  |  |  |
| Reading Strategies (RS) | RS1 | V16 |
|  | RS2 | V17 |
|  | RS3 | V18 |
|  | RS4 | V19 |
|  | RS5 | V20 |
|  | RS6 | V21 |
|  | RS7 | V22 |
|  | RS8 | V23 |
|  | RS9 | V24 |
|  |  |  |
| Perceived Benefits of Reading (PBR) | PBR1 | V37 |
|  | PBR2 | V38 |
|  | PBR3 | V39 |
|  | PBR4 | V40 |
|  | PBR5 | V41 |
|  | PBR6 | V42 |
|  | PBR7 | V43 |
|  | PBR8 | V44 |
